# Versatility of Retzius-Sparing Prostatectomy: Its Application in Renal Transplant Patient and En-bloc Abdominal-Perineal Resection

**DOI:** 10.1245/s10434-021-10804-6

**Published:** 2021-09-26

**Authors:** Chi Hang Yee, Sze Jin Adrian Yu, Man-Fung Ho, Kaori Futaba, Tony Mak, Jeremy Yuen Chun Teoh, Peter Ka Fung Chiu, Simon Siu-Man Ng, Chi-Fai Ng

**Affiliations:** 1 S.H. Ho Urology Centre, Department of Surgery, Prince of Wales Hospital, The Chinese University of Hong Kong, Shatin, Hong Kong; 2 Division of Colorectal Surgery, Department of Surgery, Prince of Wales Hospital, The Chinese University of Hong Kong, Shatin, Hong Kong

The Retizius-sparing (RS) approach to prostatectomy has several proposed advantages over the conventional retropubic approach. This includes reduced post-prostatectomy stress urinary incontinence rates and better preservation of potency.^1–3^ While a smaller workspace may render this technique more technically challenging, this approach provides an advantage beyond functional outcome in certain clinical situations. We present two scenarios where Retzius-sparing robotic-assisted radical prostatectomy (RS-RARP) provided additional versatility that was translated into a more desired clinical outcome.

The first case was a 76-year-old renal transplant patient diagnosed with intermediate-risk prostate cancer. His well-functioning low-lying graft kidney in the left lower quadrant was in close relation to the prostate gland, precluding either conventional retropubic prostatectomy or radiotherapy as a treatment option. RS-RARP was performed for the patient, yielding satisfactory oncological and functional outcomes. The second case was a 61-year-old patient who had a rectal tumour with suspected prostate invasion on MRI. After neoadjuvant chemotherapy and radiotherapy, robotic en-bloc abdominal-perineal resection of the rectum, using a Retzius-sparing prostatectomy technique, was performed to save the patient from total exenteration and double stomata. A modified RS-RARP was adopted to keep the surgical plane between the rectum and prostate. Both procedures were uneventful.

In conclusion, the Retzius-sparing approach is a versatile technique which helps the surgeon to conduct a prostatectomy when special anatomical considerations are at play.

## Supplementary Information

Below is the link to the electronic supplementary material.

Supplementary file1 (MP4 260251 kb)

Supplementary file2 (DOCX 25 kb)

## References

[1] Dalela D, Jeong W, Prasad MA (2017). A pragmatic randomized controlled trial examining the impact of the Retzius-sparing approach on early urinary continence recovery after robot-assisted radical prostatectomy. Eur Urol..

[2] Galfano A, Di Trapani D, Sozzi F (2013). Beyond the learning curve of the Retzius-sparing approach for robot-assisted laparoscopic radical prostatectomy: Oncologic and functional results of the first 200 patients with ≥1 year of follow-up. Eur Urol..

[3] Lim SK, Kim KH, Shin TY (2014). Retzius-sparing robot-assisted laparoscopic radical prostatectomy: Combining the best of retropubic and perineal approaches. BJU Int..

