# Supplementary material for: Versatility of Retzius-Sparing Prostatectomy: Its Application in Renal Transplant Patient and En-bloc Abdominal-Perineal Resection
Source: Ann Surg Oncol. 2021 Sep 26;29(2):1486–7. doi: 10.1245/s10434-021-10804-6 (PMC8724161; doi:10.1245/s10434-021-10804-6)
Supplement: Supplementary file 2 — Supplementary file2 (DOCX 25 kb) [file 10434_2021_10804_MOESM2_ESM.docx]

0’00”

In this video, we are going to present the application of a Retzius-sparing prostatectomy technique under complex surgical conditions, namely a renal transplant patient and en-bloc abdominal-perineal resection.

0’15”

The conventional radical prostatectomy technique would involve detaching the bladder from the anterior abdominal wall, accessing the prostate through the retropubic route.

0’27”

For the Retzius-sparing technique, the peritoneum is incised at the seminal vesicle level to access the prostate, leaving the attachment between the bladder and the anterior abdominal wall intact. A randomized-controlled trial by Mani Menon showed that this technique allows an earlier recovery of continence function when compared with the conventional retropubic technique. This video demonstrates that the Retzius-sparing technique can be a versatile approach, allowing access to the prostate in challenging situations.

0’56”

This gentleman with prostate cancer had a renal transplant performed 10 years ago, and the MRI demonstrates a low-lying graft kidney in close proximity to the prostate gland.

1’08”

Because of this low-lying graft kidney, radiotherapy to the prostate gland was deemed unsafe by oncologists, and it made access to the prostate via the conventional retropubic approach difficult.

1’18”

As a result, a robotic Retzius-sparing prostatectomy technique was used. Intra-abdominally you can appreciate the relationship between the graft kidney in the left lower quadrant and the bladder with a urethral Foley inserted.

1’31”

Incision of the peritoneum was made at the seminal vesicle level, and subsequently the vasa were transected and the seminal vesicles were delivered.

1’47”

Posterior dissection between the prostate and rectum was performed in a conventional prostatectomy manner until the apex.

1’59”

When the posterior plan had been fully developed, the lateral vascular pedicles were divided between surgical clips in order to allow lateral dissection between the prostate gland and pelvic side wall.

2’18”

Here, the prostate was dissected away from the right pelvic side wall, and the dissection continued to the apex, and anteriorly just below the deep venous complex.

2’30”

A similar effort was attempted on the left side where the graft kidney was located. However, a lot of adhesion was encountered, making the dissection between the left lateral prostate wall and pelvic side wall quite difficult.

2’49”

As a result, a modified technique was adopted. The bladder neck was identified and transected before the left lateral prostate wall was completely freed.

3’12”

Once the bladder neck was opened, we could peep into the prostatic urethra mucosa. An incision into the posterior lip of the bladder neck was made, and subsequently the bladder neck transection was completed.

3’32”

The prostate was further dissected away from the anterior and left lateral attachment, going in the direction of the apex.

3’42”

In the end, the urethra was exposed and transected. The specimen was delivered.

3’54”

An anastomosis was made between the bladder and the urethra using 3/0 V-loc suture, with the bladder hanging at the 12 o’clock position.

4’07”

Eventually, the peritoneal incision was closed.

4’14”

The operation took 223 min with a blood loss of 250 ml. The Foley catheter was taken off on post-operative day 7, and the patient achieved immediate continence without the need of any pad. The pathology came back as a pT2c Gleason score 3+4 prostate cancer. At the 9-month follow-up, the patient had achieved an undetectable PSA level.

4’37”

The next patient was a gentleman with rectal cancer who had undergone neoadjuvant chemotherapy and radiotherapy. After neoadjuvant treatment, MRI found the rectal tumour with suspected prostate invasion.

4’49”

While the patient warranted an abdominal-perineal resection, the challenge was to preserve the native bladder so as to avoid a total exenteration procedure with double stomata. Furthermore, if the bladder could be left attached to the anterior abdominal wall during the procedure, it would make dissection of the rectum more convenient without an obscured view.

5’08”

As a result, an en-bloc abdominal-perineal resection with Retzius-sparing prostatectomy was performed.

5’19”

The working ports setting was similar to conventional robotic rectal surgery, as show in the diagram. Two assistant ports of 5 mm and 10 mm were inserted in the upper abdomen for the bedside surgeon. The patient was positioned in a Trendelenburg plus left-side-up configuration, as shown in the picture. The lower limbs were in the Lloyd-Davies position to allow access to the perineum later.

5’40”

At the beginning of the procedure, the rectosigmoid junction was mobilized and a total mesenteric excision was performed.

5’52”

Then the peritoneum was incised at the seminal vesicle level, like before, trying to access the prostate behind the bladder. One modification of the Retzius-sparing technique in this case was that the posterior plane between the prostate and the rectum was left untouched. The prostate was mobilized only laterally and anteriorly.

6’14”

We proceeded with the left lateral dissection of the prostate with it still sitting on top of the rectum.

6’32”

Then we moved to the right lateral wall dissection, exposing the prostate capsule on the right side.

6’51”

Once the dissections on both lateral walls were completed, the bladder neck was identified and transected.

7’08”

When the bladder neck was opened, the Foley catheter was pulled away from the bladder.

7’17”

A further incision into the posterior lip of the bladder neck completed the transection, and dissection of the prostate was continued behind the deep venous complex until we had reached the urethra.

7’31”

The urethra was transected, and the prostate was left attached to the rectum.

7’49”

After the perineal part had been completed, the whole specimen was removed.

8’00”

The bladder neck was sutured back to the urethra while the perineal part was being closed. Continuity of the urinary tract was restored.

8’27”

The operation took 382 min with a blood loss of 200 ml. The final pathology did not reveal any residual tumour. At the 9-month follow-up, the patient had persistent stress urinary incontinence that required pelvic floor exercise.

8’42”

In conclusion, in complex pelvic conditions a retropubic prostatectomy approach may encounter challenges. A Retzius-sparing prostatectomy technique can provide an alternative for accessing the prostate in these challenging situations. Thank you.
